# Catechin-Induced changes in PODXL, DNMTs, and miRNA expression in Nalm6 cells: an integrated in silico and in vitro approach

**DOI:** 10.1186/s12906-024-04521-2

**Published:** 2024-06-15

**Authors:** Ali Afgar, Alireza Keyhani, Amirreza Afgar, Mohamad Javad Mirzaei-Parsa, Mahdiyeh Ramezani Zadeh Kermani, Masoud Rezaei, Mohammad Ebrahimipour, Ladan Langroudi, Mahla Sattarzadeh Bardsiri, Reza Vahidi

**Affiliations:** 1 Research Center for Hydatid Disease in Iran, Kerman University of Medical Sciences, Kerman, Iran; 2 Leishmaniasis Research Center, Kerman University of Medical Sciences, Kerman, Iran; 3 Department of Hematology and Medical Laboratory Sciences, Faculty of Allied Medicine, Kerman University of Medical Science, Kerman, Iran; 4 Department of Immunology, School of Medicine, Kerman University of Medical Sciences, Kerman, Iran; 5 Stem Cells and Regenerative Medicine Innovation Center, Kerman University of Medical Sciences, Kerman, Iran; 6 Student Research Committee, Faculty of Allied Medicine, Kerman University of Medical Sciences, Kerman, Iran

**Keywords:** PODXL, Methyltransferase, miRNAs, Docking, MD simulation, ALL, And catechin

## Abstract

**Background:**

This study explored the impact of predicted miRNAs on DNA methyltransferases (DNMTs) and the *PODXL* gene in Nalm6 cells, revealing the significance of these miRNAs in acute lymphocytic leukemia (ALL).

**Methods:**

A comprehensive approach was adopted, integrating bioinformatic analyses encompassing protein structure prediction, molecular docking, dynamics, and ADMET profiling, in conjunction with evaluations of gene and miRNA expression patterns. This methodology was employed to elucidate the therapeutic potential of catechin compounds in modulating the activity of DNA methyltransferases (*DNMTs*) and the *PODXL* gene.

**Results:**

The findings from our investigation indicate that catechins possess the capability to inhibit DNMT enzymes. This inhibitory effect is associated with the upregulation of microRNAs miR-200c and miR-548 and a concurrent downregulation of *PODXL* gene expression. These molecular interactions culminate in an augmented apoptotic response within ALL (Nalm6) cells.

**Conclusion:**

The study posits that catechins may represent a viable therapeutic avenue for inducing apoptosis in ALL cells. This is achieved through the modulation of epigenetic mechanisms and alterations in gene expression profiles, highlighting the potential of catechins as agents for cancer therapy.

**Supplementary Information:**

The online version contains supplementary material available at 10.1186/s12906-024-04521-2.

## Introduction

ALL is one of the most common cancers in children and is occasionally observed in adults. ALL is a malignant bone marrow disease in which primary lymphoid precursors proliferate and replace normal bone marrow hematopoietic cells [1]. Currently, molecular changes associated with the pathogenesis of leukemia are being studied and identified. These changes are used as diagnostic markers to minimize false-negative results and enable timely diagnosis of the disease before it progresses to metastatic stages. This attention is aimed at identifying appropriate treatment options. Among the molecular alterations involved in the disease process, the expression of genes and epigenetic factors plays a significant role [2]. A wide range of genes that promote apoptosis, unlimited cell growth, angiogenesis, and tumor metastasis have been identified. These genes include *NPMI, WTI, BAALC*, and *FLT3* [3]. Another important gene is *the PODXL* gene, which is upregulated in a wide range of cancers, including malignant brain tumors; breast, prostate, testicular, liver, pancreas, and kidney cancers; and leukemia. Additionally, research has demonstrated that the expression of this protein is associated with severe malignancy, poor prognosis, and metastasis [4]. The expression of PODXL in ALL is significant for several reasons. These included the expression of proteins associated with PODXL and CD34 in most leukemic blasts and the expression of PODXL in normal precursor cells. Additionally, the expression of the PODXL transcriptional regulator Wilms’ tumor I is observed in many blast cells of ALL and acute myelocytic leukemia [5]. These new findings highlight the role of PODXL in survival, migration, cell proliferation, drug resistance development, and metabolic reprogramming in non-Hodgkin lymphoma [6]. Therefore, considering the significant role of *PODXL* in the development of acute leukemia, invasion, and metastasis, the expression level of PODXL has been regarded as a diagnostic and prognostic factor [7]. In this regard, identifying the factors involved in the effective expression of this gene will be very helpful. One of these factors is microRNAs. MicroRNAs are endogenous, single-stranded, small 20–23 noncoding RNAs that regulate the expression of approximately 60% of genes encoding proteins at the posttranscriptional level by binding to 3’UTRs. These molecules affect the expression of mRNAs, causing them to degrade or preventing their translation [8, 9]. Each miRNA can regulate the expression of numerous target genes and can be controlled by several other miRNAs. Therefore, miRNAs can affect the expression of the *PODXL* gene, and changes in miRNA expression determine the expression of this gene. Among the studies conducted in this field, one study demonstrated an inverse association between miR-125b and the *PODXL* gene in umbilical artery endothelial cells and aortic smooth muscle cells (HAVSMCs), suggesting that inhibiting miR-125b to reduce PODXL expression could be considered a treatment option for atherosclerosis [10]. Furthermore, abnormal expression of the podocalyxin gene in acute myelocytic leukemia is associated with a decrease in miR-199b [11]. As a result, the use of this miRNA can serve as both a therapeutic and prognostic tool for this type of cancer. In this context, additional epigenetic mechanisms, such as methylation, play important roles in regulating these genes. Abnormal DNA methylation is a prominent feature of ALL, and numerous studies indicate that it can play a significant role in the development and progression of ALL [4]. Abnormal epigenetic regulation, particularly gene promoter DNA hypermethylation, is a recurring gene silencing mechanism associated with disease prognosis and treatment response in patients with B-cell progenitors (ALL-B). Studies on ALL leukemia have shown that the expression of several microRNAs is decreased, and their levels can be restored by treatment with methyltransferase inhibitors, such as zebularine [12]. The establishment and maintenance of DNA methylation patterns are critical epigenetic mechanisms, orchestrated by the collaborative functions of DNA methyltransferases, namely DNMT1, DNMT3A, and DNMT3B. DNMT1 predominantly acts as a maintenance methyltransferase, faithfully propagating methylation signatures post-DNA replication. Conversely, DNMT3A and DNMT3B are primarily involved in de novo methylation, laying down new methylation patterns during early developmental stages and in response to environmental cues [13, 14].

Catechins, particularly epigallocatechin-3-gallate (EGCG), are polyphenolic compounds with significant bioactivity, including apoptosis induction via caspase activation, Bcl-2 family protein modulation, and interference with cell survival pathways. Additionally, catechins are implicated in epigenetic alterations, affecting DNA methylation and histone modification patterns [15]. Green tea catechins, potent polyphenols, exhibit anti-cancer properties across cancer types, including myeloid and lymphoid leukemias, in both in vitro and in vivo settings. The therapeutic efficacy of these catechins is dose-dependent and time-sensitive [16, 17].

The integration of computational methods with traditional herbal medicine research is revolutionizing the discovery and investigation of bioactive compounds. Recent studies have utilized computational techniques to explore the therapeutic advantages and action mechanisms of various herbal extracts, demonstrating the potential of these methods in predicting interactions, identifying target receptors, and revealing the biological activities of herbal compounds. For instance, a study conducted by Obaidullah and colleagues in 2021 utilized computational docking experiments to investigate the neuropharmacological effects of Cnesmone javanica Blume leaf extract, suggesting potential anxiolytic and depressive activities [18]. Similarly, Rahman and colleagues in 2020 used computational docking to study the antidepressant effects of compounds found in Cycas pectinata, highlighting their potential as therapeutic agents [19].

In the realm of antimicrobial research, a study by Emran in 2015 demonstrated the efficacy of Bacopa monnieri leaf extract against Staphylococcus aureus through computational docking [20]. In 2021, Amin synthesized MGP analogs and identified their potential as antiviral agents against COVID-19 using molecular docking [21]. Furthermore, a study by Munia in 2023 synthesized uridine derivatives and conducted computational tests to analyze their antibacterial and anticancer properties, yielding promising results [22].

In the present study, we conducted a comprehensive analysis of microRNA (miRNA) expression profiles and the functional implications of the *PODXL* gene post-catechin treatment, utilizing cutting-edge bioinformatics tools. Our investigation included the prediction of novel miRNAs through an in-depth examination of the 3’UTRs of key methylation-related genes, specifically *DNMT3B, DNMT3A, and DNMT1*. By integrating bioinformatics approaches with empirical techniques, we were able to ascertain the impact of catechin on the regulation of miRNA expression, as well as elucidate the roles of *PODXL* and *DNMTs* family genes in this context.

## Materials and methods

### Availability of sequences and BLAST queries

To identify suitable structures for our study, we retrieved the amino acid sequences of DNMT1, DNMT3A, and DNMT3B from the NCBI protein database (https://www.ncbi.nlm.nih.gov/protein/) in FASTA format. We used these sequences as queries in a BLAST search against ‘Protein Data Bank proteins’, which revealed experimentally confirmed structures for each target protein. This process facilitated the selection of an appropriate template structure for investigating interactions with catechin compounds.

### Prediction of protein secondary structure and topology

The secondary structures of DNMT1, DNMT3A, and DNMT3B were determined using the SOPMA server (https://npsa-prabi.ibcp.fr/NPSA/npsa_sopma.html). Concerning the neural network, SOPMA can predict a significant portion of the amino acids involved in the secondary structure, ultimately leading to the generation of 3D models from the 2D structures.

### The physicochemical characteristics determined from the sequences

The structural and functional characteristics of a protein can be estimated by analyzing its physical and chemical properties. To determine these characteristics, the protein structure sequence was submitted to the Prot Param web server (https://web.expasy.org/protparam/).

### Prediction of functional pockets and residues

The online service HotSpot Wizard 3 (https://loschmidt.chemi.muni.cz) was used to predict the functional amino acids of the proteins DNMT1, DNMT3A, and DNMT3B. To obtain the core structural pockets and cavities, the CASTp web server at http://sts.bioe.uic.edu/castp/index.html?2cpk was used. The output of the CASTp server provides measurements in angstroms, ranging from 0.0 to 10.0. The prepared protein structure was then utilized for the docking process, and grid boxes were defined for each target protein. The coordinates for the grid boxes were set as follows (in Å): DNMT1 (x = 30, y = 24, z = 36), DNMT3A (x = 19, y = 30, z = 29), and DNMT3B (x = 30, y = 30, z = 30).

### Prediction of the ADMET of chemical compounds

For a drug to be considered suitable, it must possess favorable biochemical activity, pharmacokinetics, and safety, as well as high potency and selectivity. Additionally, it should meet the criteria for ADMET (Absorption, Distribution, Metabolism, Excretion, and Toxicity). An ideal drug must be able to distribute itself effectively into various tissues and organs, undergo metabolism without an immediate decrease in activity, and be excreted from the body properly [23]. Due to the incomplete medicinal properties of catechin in the DrugBank database, it was subjected to evaluation using the ADMETlab 2.0 server (https://admetmesh.scbdd.com/service/evaluation/index) to determine its physicochemical, medicinal chemistry, and ADMET parameters [24].

### Energy minimization of proteins and ligands

Energy minimization is crucial for the accurate determination of molecular spatial arrangement. In protein system modeling, adjusting the hydrogen bond networks is essential for eliminating disruptive contacts and minimizing the overall system energy, taking into account components such as stretching, bending, and torsional potential energy [25]. The YASARA server (http://www.yasara.org/minimizationserver.htm) used the Amber force field to minimize the energy needed for DNMTs and catechin compounds. Its optimized energy functions resulted in superior structural models, leveraging the minimal energy of empirical structural models [26].

### Docking of catechin and methyltransferases

The HDOCK web server (http://hdock.phys.hust.edu.cn) and AutoDock4 were utilized to investigate the interactions between catechin and DNMT proteins [27]. HDOCK employs a hybrid approach that combines template-based modeling and ab initio-free docking to achieve protein-protein and protein-d-DNA/RNA docking. Moreover, AutoDock4 is an integrated platform for predicting protein-ligand interactions using a Lamarckian genetic algorithm. Open Babel software was used to convert the necessary file formats for the server and program [28]. The molecular Docking protocol was initiated by detecting pockets/cavities in the protein structure using the CastP server with a radius probe set to 1.4 Å. Subsequently, the 3D structure of the protein was optimized using the Dockprep command in Chimera software. This optimization process involved the removal of solvent, co-crystallized ligand, and non-complex ions, followed by loop modeling to refine the structure and the addition of hydrogen ions.

### Two-dimensional interaction diagram

To identify the amino acids involved in protein-protein interactions, a 2D interaction plot was generated for the inhibitory chemical catechin with the DNMT1, DNMT3A, and DNMT3B proteins. The computations were performed using LigPlot + software, accessible at https://www.ebi.ac.uk/thornton-srv/software/LigPlus/. Protein-ligand interactions were also analyzed using Discovery Studio software, which is built upon the SciTegic Enterprise Server, an open operating platform. This tool also provides the possibility to analyze other aspects related to protein-ligand interactions.

### Molecular dynamics simulations

The superior Docking results of catechin with the DNMT1 and DNMT3B proteins, which exhibited significant changes before and after treatment, were subjected to MDs using the CHARMM 27 all-atomic force field. The protein-ligand complex was solvated in a triclinic box using periodic boundary conditions and the TIP3P water model. The Na + and Cl- ions were added to neutralize the system. The SwissParam server was used to determine the ligand parameters and topology. The internal constraints of the protein-ligand complex were relaxed by 50,000 steps of steepest descent energy minimization, leading to restriction of the positions of all heavy atoms. Before the MDs, the systems were heated using a V-rescale thermostat to obtain a temperature of 300 K with 0.1 ps as the coupling constant, and equilibration was achieved in NVT. Then, the solvent density was sustained using a Parrinello-Rahman barostat with a pressure of 1 bar, a coupling constant of 0.1 ps, and a temperature of 300 K to obtain equilibration in the NPT by gradually discharging the restraint on heavy atoms step by step. Finally, an MD was performed for the complexes for 40 ns with an integration time step of 2 fs. Finally, trajectory analyses, such as RMSD, RMSF, Rg, SASA, and H-bonds of protein-ligand complexes, were performed using the GROMACS package.

### miRNA and target mRNA prediction

To identify target genes, miRDB, RNAhybrid, PICTAR4, DIANAmT, miRWalk, miRanda, DIANAmT, RNAhybrid, PITA, RNA22, PICTAR5, and TargetScan software were used for predicting target microRNAs. In the process of identifying the most promising microRNA (miRNA) candidates, several criteria were meticulously applied. These included the extent of the seed region’s complementarity to the target mRNA, the conservation of seed pairing, the concordance among various miRNA target prediction software, and the concurrent targeting of DNMT genes within the 3’ untranslated region (3’UTR). Subsequent to this rigorous selection process, one miRNA was distinguished from the pool of potential candidates, predicated on its specific localization within the 3’UTR of the DNMT gene, thereby qualifying it for further analysis.

### Special probes, primers, and stem‒loop design for the expression of MicroRNAs

First, to generate predicted miRNAs for all DNMT genes, the microRNA sequences of interest were obtained from the miRBase database by completing the registration process at www.mirbase.org. The specific miRNAs targeted were miR-548, miR-200c, miR-193a, and miR-148a-5p. To determine the smallest detectable number and ensure high sensitivity for these target miRNAs in the sample, we utilized the stem-loop sequence published by Faridi et al. [29]. The stem-loop design includes a 6-nucleotide sequence at the end, which is complementary to the 3’ end region of each microRNA, allowing for specific detection of each miRNA. For the forward primers, most mature miRNAs were utilized, with minor modifications to the 5’ primer. To evaluate primer specificity, the BLAST primer page was used at https://٫www.ncbi.nlm.nih.gov٫tools٫primer-blast٫ on the NCBI website was utilized. The Tm values of the primers and probes were adjusted using Gene Runner v. 6.5.52 software (Frank Buquicchio and Michael Spruyt.) following the standard conditions of real-time PCR. The specificity of each miRNA was confirmed by real-time PCR amplification of the target sequence using cDNA from the miRNA stem loops. Finally, relative expression and/or fold change analyses were conducted by comparing the CT values of the target miRNAs to those of the U6 reference gene, as shown in Tables 1 and 2.

**Table 1:** Designed primers, probes, and RT Stem-loops

miRNA	Accession number	RT specific stem‒loop primer
RT-primer miR-548	MIMAT0031890	GTATGCGGCTACCCTCGGACCCTGCTTAGTGCCATGCCTGCCATCGGAGCCGCATAC*AAAGTA*
RT-primer miR-200c	MI0000650	GTATGCGGCTACCCTCGGACCCTGCTTAGTGCCATGCCTGCCATCGGAGCCGCATAC *TCCATC*
RT-primer miR-193a	MI0000487	GTATGCGGCTACCCTCGGACCCTGCTTAGTGCCATGCCTGCCATCGGAGCCGCATAC *ACTGGG*
RT-primer miR-148a-5p	MIMAT0004549	GTATGCGGCTACCCTCGGACCCTGCTTAGTGCCATGCCTGCCATCGGAGCCGCATAC*AGTCGG*
RT primer U6	NR_004394.1	GTATGCTGCTACCTCGGACCCTGCTTAGTGCCATGCCTGCCATCGAGCAGCATAC *CGAATT*
F- miR-548	MIMAT0031890	CCCGCAAAAACTGCAGTTACTTT
F- miR-200c	MI0000650	TAATACTGCCGGGTAATGATGGA
F- miR-193a	MI0000487	AATGGCCTACAAAGTCCCAGT
F- miR-148a-5p	MIMAT0004549	AAAGTTCTGAGACACTCCGACT
F-U6	NR_004394.1	GCAAGGATGACACGCAAATT

**Taq man probe**: FAM 5’AGTGCCATGCCTGCCATCGAGC 3’ BHQ-1

**Universal reverse:5’** GCTGCTACCTCGGACCCT 3’;

*miRNA complementary specific sequences are underlined*

**Table 2:** Primer sequences for real-time PCR

Gene	Accession number	Primer 5’-3’
F-DNMT3A	NM_001130823.3	AACAGGCCGTTGGCATCC
R-DNMT3A	NM_001130823.3	GTAATGGTCCTCACTTTGCTGAAC
F- DNMT1	NM_001375819.1	TTATCCGAGGAGGGCTACCTG
R- DNMT1	NM_001375819.1	TCCCGGTTGTAAGCATGAGC
F-DNMT3B	NM_175848.2	GACTTGACAGGCGATGGCG
R-DNMT3B	NM_175848.2	CTGTTGTTATTTCGAGTTCGGACA
F-reference gene (UBE2D2)	NM_181838.2	AGAATCCACAAGCTCCCTCC
R-reference gene (UBE2D2)	NM_181838.2	TGCCACCCAAGAGGTAAGTG
F- PODXL	XM_034965004.1	ACGAGAGTAACTGGGCAAAGTG
R- PODXL	XM_034965004.1	GTGAAGGTGGCTTTGACTGC

### Cell lines and drugs

Catechin, with a purity greater than 95%, was generously provided as a gift by Reza Fotouhi Ardakani (Qom University of Medical Sciences, Iran). The Nalm6 cell line was acquired from the Institute Pasteur of Iran’s cell bank, and peripheral blood cells (PBCs) were isolated utilizing proprietary techniques within our research facility. Subsequent to procurement, all cells were cultured in RPMI 1640 medium supplemented with 15% fetal bovine serum, 2 mM glutathione, 100 units/mL of penicillin, and 100 µg/mL of streptomycin (kalazist, Iran, batch-73,041). Upon attaining 70–80% confluency, the cells were meticulously transferred to a 6-well cell culture plate for further experimentation.

### MTT assay

Initially, 2 × 10^4^ cells were seeded in a 96-well plate in a volume of 100 µl and incubated. Then, the Nalm6 cells were treated with different concentrations of catechins (0, 2.5, 5, 10, 20, 40, 60, 80, or 110 µM) [30, 31] for 24 h. Next, MTT dye was added to the sample to a final concentration of 0.45 mg/mL, 100 µl of DMSO was added to each well, and the solution was mixed. Finally, the absorbance of the sample was measured at 570 nm to calculate the IC50 using Prism 8.0.2 software. The rate of cell proliferation inhibition was determined using the formula [1-(OD value of compound ٫OD value of the control)] 100%.

### Catechin-induced morphological alterations

A total of 1 × 10^6^ Nalm6 cells/ml were seeded in 12-well plates. After treatment with different concentrations of catechin (0, 10, 15, or 20 µM), the morphology of the cells was evaluated under a microscope [31] for 24 h. Subsequently, the samples were stained with DAPI (20 mM) to investigate the impact of different concentrations of catechins on the cytoplasmic morphology of the target cells.

### Annexin V and propidium iodide flow cytometry assay

To achieve this objective, a total of 5 × 10^5^ Nalm6 cells were subjected to treatment with the “IC50” compound, specifically 35 µM, in 6-well plates for 24 h. After incubation, the cell pellet was isolated by centrifugation and then washed with PBS. Afterward, the cells were exposed to 500 µl of 1× binding buffer. Subsequently, 5 µl of annexin V was added to the samples, which were subsequently allowed to incubate in the dark for 10 min. Next, 5 µl of Pl dye was added, and the mixture was incubated for 10 min in the dark. Flow cytometry was then used to measure the percentage of phosphatidylserine released on the cell surface, and the results were analyzed using FlowJo v 7.6 software.

### Extraction of target microRNA and RNAs

To refine the extraction of target RNA, particularly microRNAs, the YTzol Pure RNA Kit (Yekta Tajhiz Azma Co., lot: AZ0211) was employed, incorporating specific modifications aimed at enhancing the purity and structural integrity of the RNA. Initially, Nalm6 cells were isolated and subjected to a brief incubation period on ice for five minutes. This was followed by the addition of 200 µl of chloroform to the cellular suspension, which was then agitated for two minutes and subsequently subjected to centrifugation at 12,000 rpm for 30 min at a temperature of 4 °C. The resultant mixture was carefully transferred into a sterile tube, and the chloroform extraction step was promptly reiterated using 100 µl of 1-bromo-3-chloropropane. Following this, the aqueous phase was decanted into a fresh tube, to which an equivalent volume of absolute ethanol was introduced. The samples were then preserved at -20 °C overnight, succeeded by centrifugation at 12,000 rpm and 4 °C for one hour. Post-centrifugation, the supernatant was discarded, and the pellet was washed with 1 ml of 70% ethanol, followed by another centrifugation at 12,000 rpm and 4 °C for 45 min. After discarding the supernatant, the RNA pellet was allowed to air-dry at ambient temperature. Subsequently, 50 µl of DEPC-treated water was added to each tube, along with 5 units of RNase-free DNase I, and the mixture was incubated for five minutes at room temperature. The DNase I was then inactivated by heating the samples for five minutes at 70 °C. The concentration and purity of the extracted RNA were ascertained using a Nanodrop 2000 spectrophotometer (Thermo Fisher Scientific, Waltham, MA, USA). The tubes containing the RNA were stored at -70 °C pending further analysis.

### cDNA synthesis and real-time PCR

cDNA was synthesized from 1000 ng of total RNA using Mu-MLV reverse transcriptase according to the kit protocol (Yekta Tajhiz, cat: YT4500). The cDNA was kept at -70 °C until analysis. The 12.5 µl PCR mixture was composed of 6.5 µl of SYBR Green master mix, 0.2 µM of each primer oligonucleotide, and 2 µl of cDNA. The real-time program was performed as follows. The initial denaturation step was 95 °C for 40 s, followed by 45 cycles of denaturation at 95 °C for 40 s and 60 °C for 20 s, and a final extension at 72 °C for 35 s. The *UBE2D2* gene was used as the internal reference gene [32]. Finally, the PCR efficiency and expression of each mRNA were assessed using LinRegPCR software. The PCR efficiency was between 95 and 108%.

### Synthesis of microRNA cDNA and quantitative real-time PCR

Following miRNA extraction, cDNA was synthesized using Mu-MLV reverse transcriptase. Four microliters of extracted miRNA, adjusted to 1200 ng of RNA, were added to 1.5 µl of stem‒loop (diluted 1.100% of the original 100 µM solution), followed by 5 µl of double-distilled water. The 10.5 µl mixture was incubated for 5 min at 65 °C in a thermocycler (Bio-Rad). Immediately, the tubes were transferred to a cold container. A mixture of 2 µl of dNTPs (10 mM) and 4 µl of 4× buffer was used. Then, 0.5 µl of RNase inhibitor (20 units), 2 µl of DTT (10 mM), and 1 µl of reverse transcription enzyme were added. cDNA synthesis was performed for 1 h at 44 °C and 10 min at 70 °C to inactivate the enzyme. The synthesized cDNA was kept at -20 °C until use. The reverse transcription products were amplified by real-time PCR. A universal reverse primer and probes with a specific primer for each miRNA were applied. Each microtube contained 6.25 µl of 2× qPCR Master Mix. The primers used were 0.74 µM reverse primer, 0.5 µM forward primer, and 0.2 µM probe for a final volume of 12.5 µl. qPCR was performed on a Rotor-Gene Q(QIAGEN). The enzyme was initially activated at 95 °C for 30 s, followed by 45 cycles of 95 °C for 15 s and 60 °C for 45 s. The *U6* gene was selected as the reference gene. The relative expression of each miRNA was statistically analyzed using the Pfafil method. P values and fold changes were calculated with GraphPad Prism software version 9.2.0 (GraphPad Software, Inc., San Diego, CA).

## Results

### BLAST search of the amino acid sequence

**Fig. 1:**
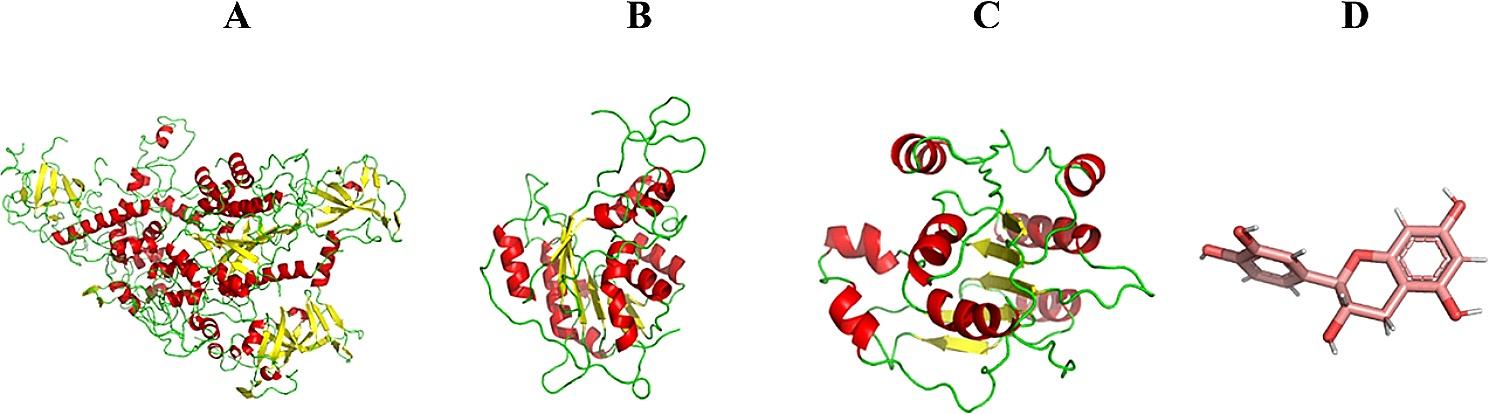
Structures of **A**: DNMT1, **B**: DNMT3A, **C**: DNMT3B, and **D**: Catechin (the structures were visualized with PyMOL software). The specific sequences for the DNMT isoforms are indexed under accession numbers NP_001124295.1, NP_072046.2, and NP_008823. Utilizing these sequences, researchers have generated several high-resolution structures. These structures are available in the Protein Data Bank (PDB) under the accession codes DNMT1 (*4WXX)*, DNMT3A (*6PA7*), and DNMT3B (*6KDA*)

The reference amino acid sequence of DNMTs can be found in the NCBI protein database, which is available at https://www.ncbi.nlm.nih.gov/. There are three accession numbers for the DNMT proteins: NP 001124295.1, NP 072046.2, and NP_008823. These accession numbers were utilized to produce several nearly complete structures with satisfactory resolution. These structures can be accessed in the PDB database using the following accession numbers: DNMT1:*4WXX*, DNMT3A:*6PA7*, and DNMT3B:*6KDA* (Fig. 1).

### Prediction of protein secondary structure and topology

**Fig. 2:**
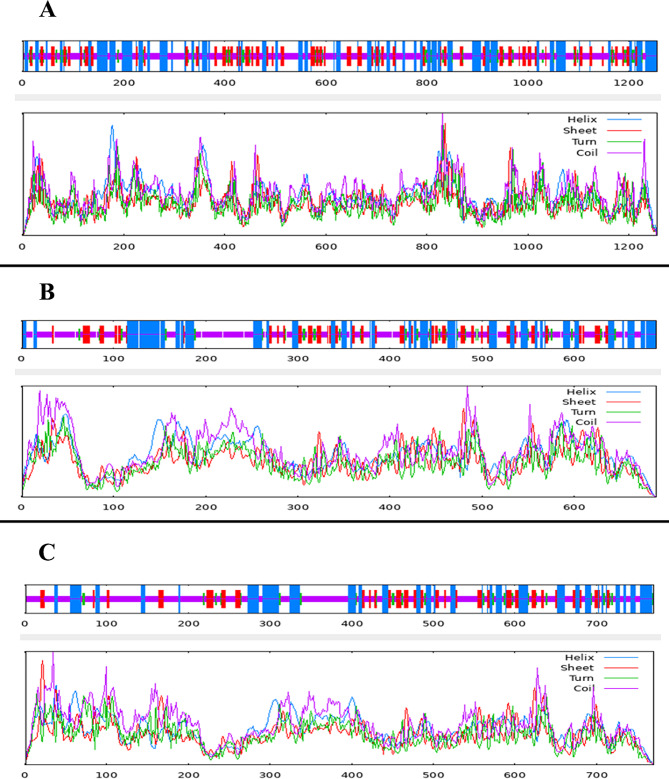
Secondary structure plot of **A**: DNMT1, **B**: DNMT3A, and **C**: DNMT3B. The structural breakdown is as follows: DNMT1: Random coils (46.50%), alpha helices (28.90%), extended strands (18.87%), and beta-turns (5.73%).DNMT3A: Random coils (46.88%), alpha helices (30.48%), extended strands (16.55%), and beta-turns (6.10%).DNMT3B: Random coils (53.25%), alpha helices (26.36%), extended strands (14.94%), and beta-turns (5.45%)

The protein secondary structure, which is crucial for Docking and Molecular Dynamics Simulations, was analyzed using the SOPMA server. Figure 2 shows that DNMT1 is composed of a random coil (46.50%), an alpha helix (28.90%), an extended strand (18.87%), and a beta-turn (5.73%). DNMT3A comprises random coils (46.88%), alpha helices (30.48%), extended strands (16.55%), and beta-turns (6.10%). The composition of DNMT3B included random coils (53.25%), alpha helices (26.36%), extended strands (14.94%), and beta-turns (5.45%). These findings offer insights into the factors influencing protein structure and function before and after docking and MD simulation.

### The extraction of physicochemical properties from the sequence

Utilizing the ProtParam server on DNMT sequences in FASTA format, DNMT1 exhibited an aliphatic index of 70.26, indicating a high proportion of aliphatic amino acids. Its GRAVY score of -0.553 implies a slightly hydrophilic nature, with an instability index of 47.52, signifying instability. The protein contains 166 negatively charged residues and 166 positively charged residues. Similarly, DNMT3A had an aliphatic index of 69.45, a GRAVY score of -0.438, and an instability index of 46.31. It has 92 negatively charged residues and 85 positively charged residues. DNMT3B displayed an aliphatic index of 63.19, a GRAVY score of -0.629, and an instability index of 58.88, indicating high instability. It includes 100 negatively charged residues and 104 positively charged residues. The Hydropathic Average (GRAVY) aids in assessing the distribution of polar and nonpolar groups within a protein’s 3D structure; this parameter is essential for pre- and post-docking, MD simulation, and 2D plot analyses.

### Functional residues and pockets in DNMTs

The functional amino acids of the DNMT1, DNMT3A, and DNMT3B proteins were determined through the use of the HotSpot Wizard web server. The identified hotspot residues within the beta chain of the DNMT1 enzyme, including CYS1226, CYS353, CYS356, CYS414, HIS418, CYS653, CYS656, CYS659, CYS664, CYS667, CYS670, CYS686, CYS691, SER1146, GLU1168, MET1169, GLY1150, LEU1151, ASP1190, CYS1191, ASN1578, and VAL1580. For DNMT3A, the hotspots were found in the K chain and included CYS710, PHE640, ASP641, SER663, GLU664, VAL665, CYS666, ASP686, VAL687, GLY707, LEU730, GLU756, ARG891, SER892, and TRP893. In the case of DNMT3B, the predicted functional amino acids were located in the L chain and included LEU651, VAL582, ALA583, SER584, GLU585, VAL586, VAL605, GLY627, GLY628, and SER629. These hotspots were mutable residues with different scores based on the web server’s scoring system, and they were situated in the catalytic pocket and/or access tunnels. The analysis also involved the identification and quantification of geometric and topological features. It was discovered that surface pockets and cavities play a role in hindering the functional development of protein targets. The methyltransferase enzymes were shown to possess several central pockets and cavities. Furthermore, the largest predicted pockets of the DNMT1, DNMT3A, and DNMT3B enzymes had solvent-accessible surface areas/volumes of 5747.91/5801.38, 531.86/480.92, and 385.82/225.80 Å2/Å3, respectively. Additionally, the following functional residues were common in the pockets of the enzymes DNMT1 and DNMT3A but not in those of DNMT3B: CYS1226, CYS356, CYS656, CYS664, CYS667, CYS670, CYS686, CYS691, SER1146, GLU1168, MET1169, GLY1150, LEU1151, ASP1190, CYS1191, ASN1578, and VAL1580. The results of the pocket, cavity, and position of the functional residues included in Table 3, docking, and 2D diagram analyses were possible.

**Table 3:** Structural and chemical pocket and cavities of castp web server

	DNMT1B		DNMT3A		DNMT3B	
	Area (Å^2^)	Volume (Å^3^)	Area (Å^2^)	Volume (Å^3^)	Area (Å^2^)	Volume (Å^3^)
**Pocket**	5747.91	5801.38	531.86	480.92	385.82	225.80
**Cavities**						
**1**	2434.66	2952.50	365.03	165.93	269.45	150.28
**2**	474.28	331.95	233.93	94.30	200.83	124.32
**3**	336.37	260.90	86.24	68.37	177.04	57.18
**4**	136.26	199.23	56.34	21.28	119.14	52.76

### Drug-based ADMET prediction

The results are summarized in Table 4. These assessments guide drug selection, ensuring desirable efficacy and safety profiles in the design and rescreening of catechin. In summary, the catechin has been able to pass most of the medicinal chemistry property criteria, such as the famous Lipinski, Pfizer, GSK, Golden Triangle rules, and quantitative estimate of drug-likeness (QED). The higher the QED score, the more drug-likeness property, The substance’s absorption capacity did not match the Caco-2 Permeability score criteria, since it scored − 6.213 for catechin. A number above − 5.15 is regarded as excellent. According to the MDCK criterion, catechin showed moderate permeability as a small molecule. Catechin demonstrated low bioavailability based on the Plasma Protein Binding (PPB) criteria when it comes to its distribution. Nevertheless, the anticipated volume of distribution (VD) fell within the optimal range (0.652 for catechin), which is regarded as ideal when it falls between 0.04 and 20 L/kg. Furthermore, catechin exhibited exceptional permeability through the blood-brain barrier, with a value < 0.3, which is regarded as remarkable. The metabolic research indicated that catechin functions predominantly as a substrate for the CYP2C9 enzyme. Concerning excretion, it has been noted that catechin is effectively removed via the renal tubules, with a clearance rate of 16.5 ml/min/kg.

**Table 4:** Calculation of catechin ADMET by ADMETlab2.0

Physicochemical property		Medicinal chemistry		Distribution		Absorption		Environmental toxicity	
Property	Value	Property	Value	Property	Value	Property	Value	Property	Value
Molecular Weight	290.08	QED	0.51	PPB	92.35%	Caco-2 Permeability	-6.213	Bioconcentration Factors	0.937
Volume	279.249	SAscore	3.344	VD	0.652	MDCK Permeability	4e-06	IGC_50_	4.412
Density	1.039	Fsp3	0.2	BBB Penetration	0.025	Pgp-inhibitor	0.007	LC_50_FM	4.788
nHA	6	MCE-18	60.0	Fu	8.351%	Pgp-substrate	0.004	LC_50_DM	5.299
nHD	5	NPscore	2.304	**Toxicity**		HIA	0.037	**Tox21 pathway**	
nRot	1	Lipinski Rule	Accepted	**Property**	**Value**	F20%	0.998	**Property**	**Value**
nRing	3	Pfizer Rule	Accepted	hERG Blockers	0.03	F30%	0.999	NR-AR	0.011
MaxRing	10	GSK Rule	Accepted	H-HT	0.099	**Metabolism**		NR-AR-LBD	0.092
nHet	6	Golden Triangle	Accepted	DILI	0.101	**Property**	**Value**	NR-AhR	0.81
fChar	0	PAINS	1 alert	AMES Toxicity	0.616	CYP1A2 inhibitor	0.393	NR-Aromatase	0.316
nRig	17	ALARM NMR	2 alerts	Rat Oral Acute Toxicity	0.43	CYP1A2 substrate	0.224	NR-ER	0.753
Flexibility	0.059	BMS	0 alerts	FDAMDD	0.146	CYP2C19 inhibitor	0.031	NR-ER-LBD	0.459
Stereo Centers	2	Chelator Rule	1 alert	Skin Sensitization	0.947	CYP2C19 substrate	0.054	NR-PPAR-gamma	0.128
TPSA	110.38	**Excretion**		Carcinogen city	0.159	CYP2C9 inhibitor	0.323	SR-ARE	0.146
logS	-2.72	**Property**	**Value**	Eye Corrosion	0.003	CYP2C9 substrate	0.827	SR-ATAD5	0.019
logP	1.213	CL	16.512	Eye Irritation	0.914	CYP2D6 inhibitor	0.139	SR-HSE	0.846
logD	1.243	^T^1/2	0.884	Respiratory Toxicity	0.117	CYP2D6 substrate	0.31	SR-MMP	0.779
						CYP3A4 inhibitor	0.371	SR-p53	0.185
						CYP3A4 substrate	0.18		

### Energy minimization of proteins and ligands

The energy values of DNMT1, DNMT3A, DNMT3B, and the catechin chemical compound before energy minimization by YASARA tools were 6597613120.10. -139468.80, 97477.50, and − 545, respectively. The scores assigned to them prior to undergoing energy minimization were as follows: -2.06, -1.34, -2.13, and − 0.22. Following energy minimization by YASARA, the energy values of DNMT1, DNMT3A, DNMT3B, and the catechin chemical compound were − 642864.90, -166608.10, -118058.20, and − 566, respectively. The score after energy minimization was − 0.61. -0.06, -0.62, and − 0.23, respectively.

### Molecular docking

**Fig. 3:**
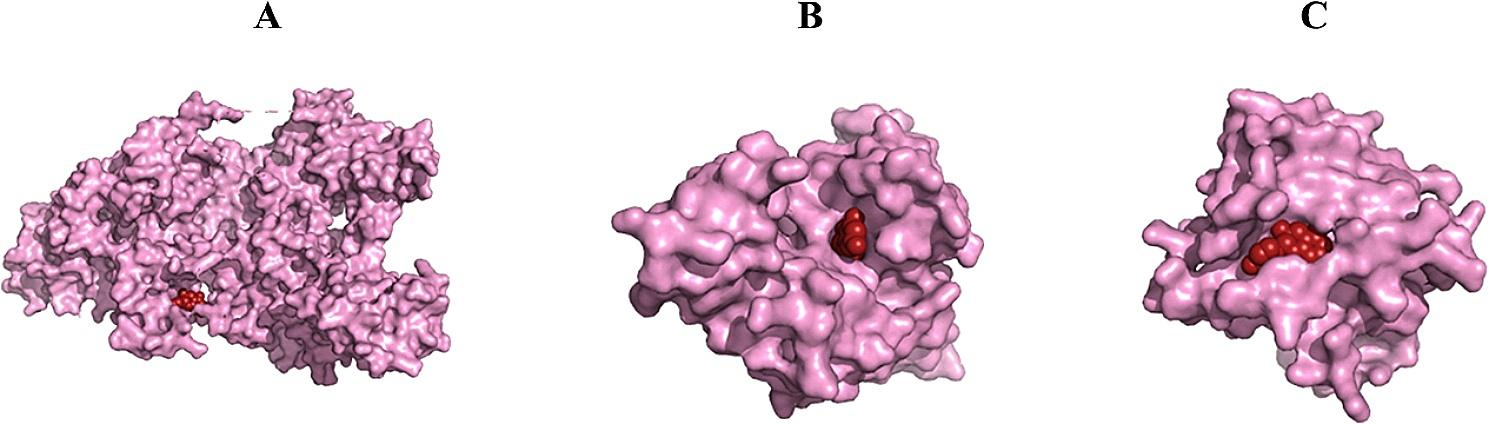
3D Docking of catechin with **A**: DNMT1, **B**: DNMT3A, and **C**: DNMT3B determined by the HDOCK server (the structures were visualized with PyMOL software). The docking procedure was executed using a genetic algorithm across 50 iterations, applying a specialized docking approach. Table 5 presents the binding energies (calculated via AutoDock4), hydrogen bond interactions, docking, and confidence scores, as well as ligand RMSD values. (sourced from the HDOCK server) for the top 10 conformations. Among these, DNMT3A exhibited the lowest binding energy, succeeded by DNMT1 and DNMT3B. Collectively, catechin demonstrated favorable binding energies with all DNMT isoforms

In this study, the chemical compound catechin was subjected to Docking analysis with DNMT1, DNMT3A, and DNMT3B using both the HDock server and AutoDock4 software. The Docking process was carried out with a genetic algorithm in 50 runs employing the specific docking method illustrated in Fig. 3. Table 5 shows the binding energy (derived from autodock4), hydrogen bonds (H-bonds), docking, and confidence scores along with ligand RMSD (from the HDOCK server) for the top 10 poses. The lowest binding energy was for DNMT3A, followed by DNMT1, and DNMT3B. overall, catechin had a reasonable binding energy for all DNMTs.

**Table 5:** Calculation of docking energy and score by AutoDock4 software and the HDOCK server

	DNMT1				
	Autodock4		HDOCK		
Pose	Binding energy (Kcal/Mol)	H-bond	Docking score	Confidence score	Ligand rmsd (Å)
**DNMT1**					
**1**	-7.92	1312B,1150B,1266B,1149B,1151	-80.2	0.1985	50.45
**2**	-7.61	463B,600B	-99.01	0.2651	35.97
**3**	-7.61	600B,462B,463B	-60.61	0.1433	48.76
**4**	-7.44	428B,424B,462B	-99.18	0.2657	51.31
**5**	-7.41	428B,424B,462B	-78.36	0.1927	53.44
**6**	-7.30	463B,595B	-108.15	0.3022	33.79
**7**	-7.23	595B,552B,1490B,553B	-93.43	0.2439	37.7
**8**	-7.15	462B	-93.14	0.2428	35.59
**9**	-7.13	428B,427B,424B(two-times),463B,597B	-87.64	0.2232	38.49
**10**	-7.00	428B,427B,424B(two times),463B	-106.55	0.2955	34.7
**Pose**	**DNMT3A**				
**1**	-9.84	893 K,643 K (two times),890 K,708 K,710 K,645 K	24.64	0.0295	360.08
**2**	-9.80	710 K,641 K,708 K,643 K,707 K	37.41	0.023	357.04
**3**	-7.73	638 K,708 K,641 K,640 K	-104.62	0.2875	360.82
**4**	-7.72	710 K,891 K,640 K,641 K,708 K	-95.54	0.2518	354.55
**5**	-7.55	(641 K,643 K),710 K	-94.77	0.2489	355.07
**6**	-7.37	893 K,710 K,643 K,645 K	-66.83	0.1593	363.78
**7**	-7.22	710 K	-129.71	0.3999	360.16
**8**	-6.39	891 K,711 K,640 K,714 K(two times)	-116.5	0.3385	363.52
**9**	-6.36	664 K,891 K,663 K	-108.76	0.3047	354.89
**10**	-6.34	663 K,891 K	-93.42	0.2439	355.45
**Pose**	**DNMT3B**				
**1**	-5.50	585 N(three times),606 N,595 N	-145.99	0.48	364.17
**2**	-5.45	606 N,585 N(two-times),595 N	-138.26	0.4416	359.94
**3**	-5.37	585 N(three times),606 N,595 N	-137.12	0.436	363.4
**4**	-5.34	586 N,585 N	-128.63	0.3947	357.04
**5**	-5.33	585 N(three times),606 N,595 N	-124.47	0.3751	350.12
**6**	-5.21	585 N(three times),606 N	-123.72	0.3715	350.17
**7**	-5.17	607 N,585 N,588 N	-120.99	0.3589	366.41
**8**	-5.17	607 N,585 N	-120.6	0.3571	357.17
**9**	-5.13	588 N,607 N	-117.14	0.3414	362.12
**10**	-5.13	607 N,585 N,588 N	-110.7	0.313	351.45

### Hydrogen bonds and hydrophobic interactions in the central pocket

The residues involved in the interaction between the main pockets of DNMT enzymes and the selected inhibitor compound, catechin, were identified through the application of the LigPlot + and Discovery Studio software. The ensuing analysis delineated specific interactions for each pose.

**Fig. 4:**
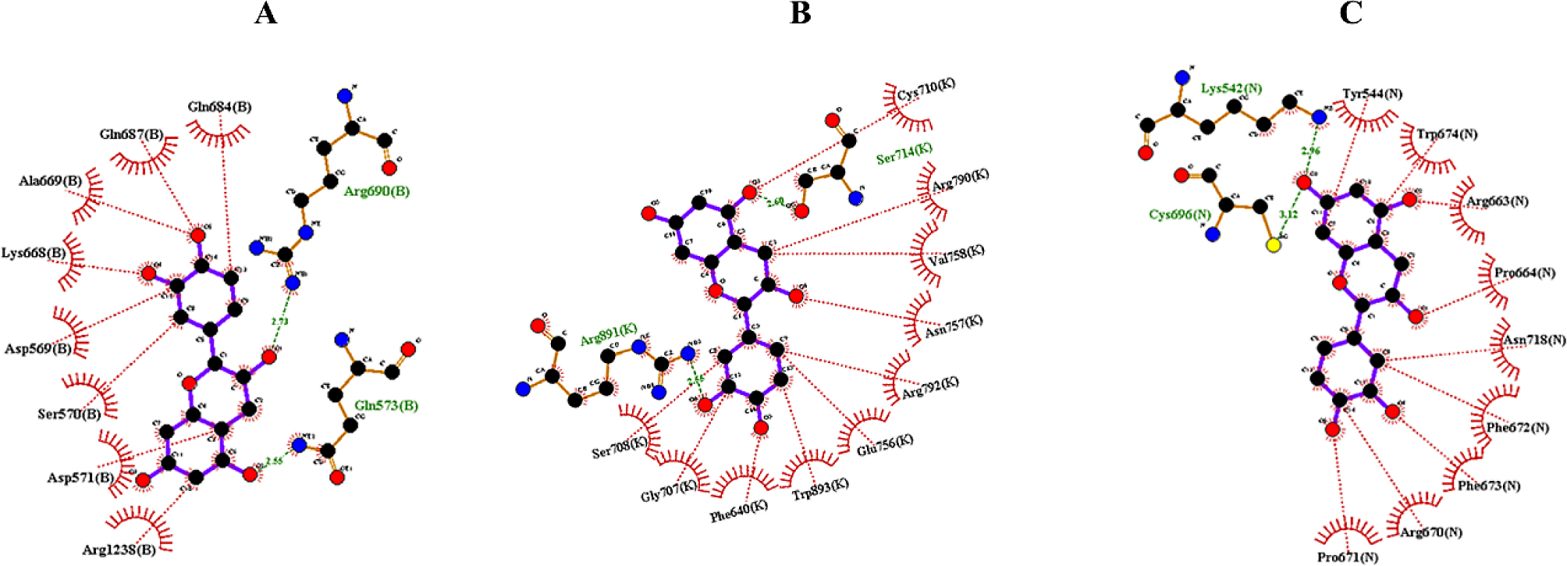
2D interactions of docking catechin with **A**: DNMT1, **B**: DNMT3A, and **C**: DNMT3B determined by Ligplot+.The two-dimensional interaction profiles of catechin docking with DNA methyltransferases DNMT1 (Figure A), DNMT3A (Figure B), and DNMT3B (Figure C), as analyzed by Ligplot+, reveal that catechin, the principal bioactive compound, is capable of establishing a variety of chemical interactions with these enzymes. These interactions encompass both hydrogen bonds and hydrophobic interactions. The multiplicity of bonding types signifies catechin’s capacity to influence the enzymatic functions of the DNMT proteins, suggesting its potential as a modulatory agent. (hydrophobic interactions are represented by green arcs, while hydrogen bonding interactions are represented by red dotted lines)

In the DNMT1 to catechin pose 6 (Fig. 4-A), four conventional hydrogen bonds were formed with GLU573, ARG69, ASP569, and GLN687. Additionally, four van der Waals interactions were observed with ASN1236, ASP571, ALA669, and SER570. Two Pi-Cation bonds were present with ARG690 and LYS668, along with one Pi-Alkyl bond involving ARG1238.

Catechin has been observed to interact with residues located in the replication focus targeting sequence (RFTS, 350–600 aa) and methyltransferase (MTase) domains (1140–1616 aa). Previous studies have demonstrated the crucial role of the RFTS domain of DNMT1 in replication-dependent DNA methylation, as well as its ability to act as an auto-inhibitory domain [33].

For DNMT3A to catechin pose 7 (Fig. 4-B), five conventional hydrogen bonds were formed with SER714, GLU756, ARG8891, GLY707, and PHE640. Additionally, three van der Waals bonds were present with VAL758, ASN757, and ARG792. Two Pi-cation and Pi-anion bonds were formed with ARG790 and GLU756. Moreover, two Pi-alkyl bonds were observed with CYS710 and ARG891. Five Pi-Donor hydrogen bonds were formed with TRP893, GLY706, PRO709, SER708, and ARG891. These interactions were similar to DNMT1, in that catechin mainly interacts with the Mtase domain (575–853 aa).

In the DNMT3B to catechin pose 1, six conventional hydrogen bonds were formed with ARG663, LYS542, CYS696, TYR544, ASN718, and PHE672. Three van der Waals bonds were present with LEU659, ARG670, and PHE673. Additionally, four Pi-Donor hydrogen bonds and carbon-hydrogen bonds were formed with PRO664, TRP674, and PRO671, as was a Pi-Sulfur bond with CYS696. Furthermore, a Pi‒Pi stacking interaction was observed with TYR544. It shows that catechin can interact with the catalytic MTase domain of DNMT3B.

Based on the analysis of two plots (Fig. 4-C), it can be concluded that catechin strongly interacts with the proteins under investigation. Overall, catechin has been found to interact with the catalytic domain of DNMT enzymes [34].

### Molecular dynamics simulation analyses

#### Root-mean-square deviation analysis

**Fig. 5:**
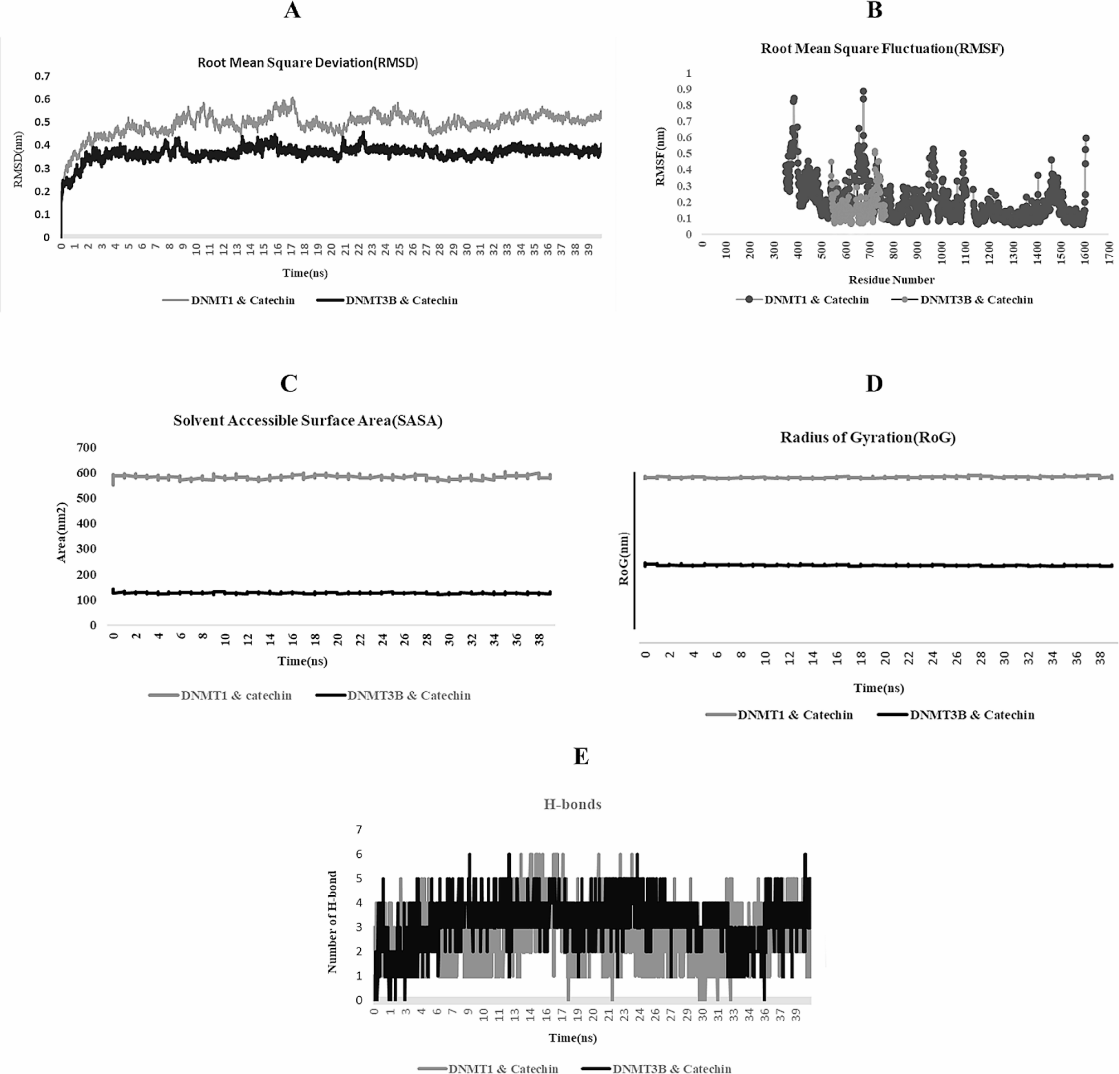
Convergence analysis of the catechin-DNMT complex MDS. Root mean square deviation (RMSD) analysis (**A**) showed that the complex reached a stable state approximately following 10 ns of simulation. Residue flexibility (RMSF) analysis (**B**): DNMT1 residues displayed high flexibility, with notable peaks at PHE676:B, LYS385:B, etc. In contrast, DNMT3B showed minimal fluctuations, suggesting greater stability. Solvent accessible surface area (SASA) analysis (**C**): The SASA values revealed DNMT3B (125.93 nm^2^) had a stronger interaction with catechin than DNMT1 (581.37 nm^2^), indicating a tighter binding with the compound. Compactness (Radius of gyration, Rg) analysis (**D**): DNMT3B exhibited a lower mean Rg deviation (1.74 nm) than DNMT1 (3.71 nm), suggesting a more compact and stable protein structure when bound to catechin. Hydrogen bonding analysis (**E**): The analysis showed more hydrogen bonds formed between DNMT3B and catechin, implying a stronger interaction and lower binding energy, indicative of a more stable complex

We analyzed the RMSD of the backbone atoms using the standard g rms function in GROMACS over a total simulation time of 40 ns. As shown in the plot in Fig. 5A, DNMT3B bound to the chemical compound catechin plateaued earlier than DNMT1. The average deviations were 0.367 nm and 0.492 nm, respectively. These findings indicated that DNMT3B combined with the catechin chemical compound was superior to DNMT1 when it was combined with the same compound.

#### Residue flexibility analysis

The conformational stability and residue flexibility of DNMT1 and DNMT3B were assessed through root-mean-square fluctuation (RMSF) analysis over a 40 nanosecond molecular dynamics simulation, utilizing the gmx_rmsf function in GROMACS. The RMSF profile, illustrated in Fig. 5B, revealed significant fluctuations at specific residues: PHE676:B, LYS385:B, LYS675:B, and GLU384:B for DNMT1; and PHE726:N, ILE725:N, ARG740:N, and LYS542:N. Notably, a pronounced peak at ARG724:N in DNMT3B was observed post-catechin interaction. These data indicate a high degree of dynamic motion and flexibility in DNMT1 residues, in contrast to DNMT3B, which displayed reduced fluctuations, suggesting enhanced stability and restricted flexibility.

#### Solvent accessible surface area analysis

The solvent-accessible surface area (SASA) of the DNMT1-catechin and DNMT3B-catechin complexes was quantified over a 40-nanosecond timeframe using the gmx sasa tool within GROMACS. As depicted in Fig. 5C, the average SASA values were determined to be 581.37 nm² for DNMT1 and 125.93 nm² for DNMT3B. The notably lower SASA value for DNMT3B suggests a more robust interaction with the catechin compound compared to DNMT1, as well as a reduced propensity for interaction with solvent molecules.

#### Compactness analysis

The radius of gyration (Rg) was calculated using the GROMACS gmx gyrate function with a simulation time of 40 ns. According to Fig. 5D, the DNMT1 and DNMT3B proteins attached to the catechin chemical compound exhibited mean Rg deviations of 3.71 nm and 1.74 nm, respectively. The radius of gyration (Rg), indicative of protein compactness, was determined for DNMT1 and DNMT3B proteins in complex with catechin using GROMACS over a 40 ns timeframe. The Rg, reflecting atom distribution around the protein’s rotational axis, serves as a proxy for structural stability. Lower Rg deviations suggest a more compact and stable protein structure, whereas higher values denote increased flexibility and spatial occupancy. Notably, DNMT1 and DNMT3B exhibited mean Rg deviations of 3.71 nm and 1.74 nm, respectively, as shown in Fig. 5D. These results imply that the catechin-bound DNMT1 assumes a less compact structure compared to DNMT3B, potentially influencing the stability and function of these proteins in a biological context.

#### Hydrogen bonding and bond distribution analysis

H-bonding analysis was performed on all protein-ligand systems during a 40 ns simulation run. The number of H-bonds was recorded using the GROMACS gmx bond tool and is shown in Fig. 5E. During the simulation period, 0.6 hydrogen bonds were formed between DNMT1 and DNMT3B via the chemical catechin compound. Furthermore, the average number of hydrogen bonds between DNMT3B and the catechin chemical compound was greater. the larger average number of hydrogen bonds represents a stronger interaction between the ligand (catechin) and the DNMT enzymes. The relationship between hydrogen bonding and binding energy is inverse. The more hydrogen bonding atoms involved in the protein-ligand interaction, the lower the binding energy. A lower binding energy signifies a stronger binding between the ligand and the protein, indicating a more potent inhibitory property for catechin against DNMT enzymes.

### Inhibitory effects of catechin on the Nalm6 cell line

**Fig. 6:**
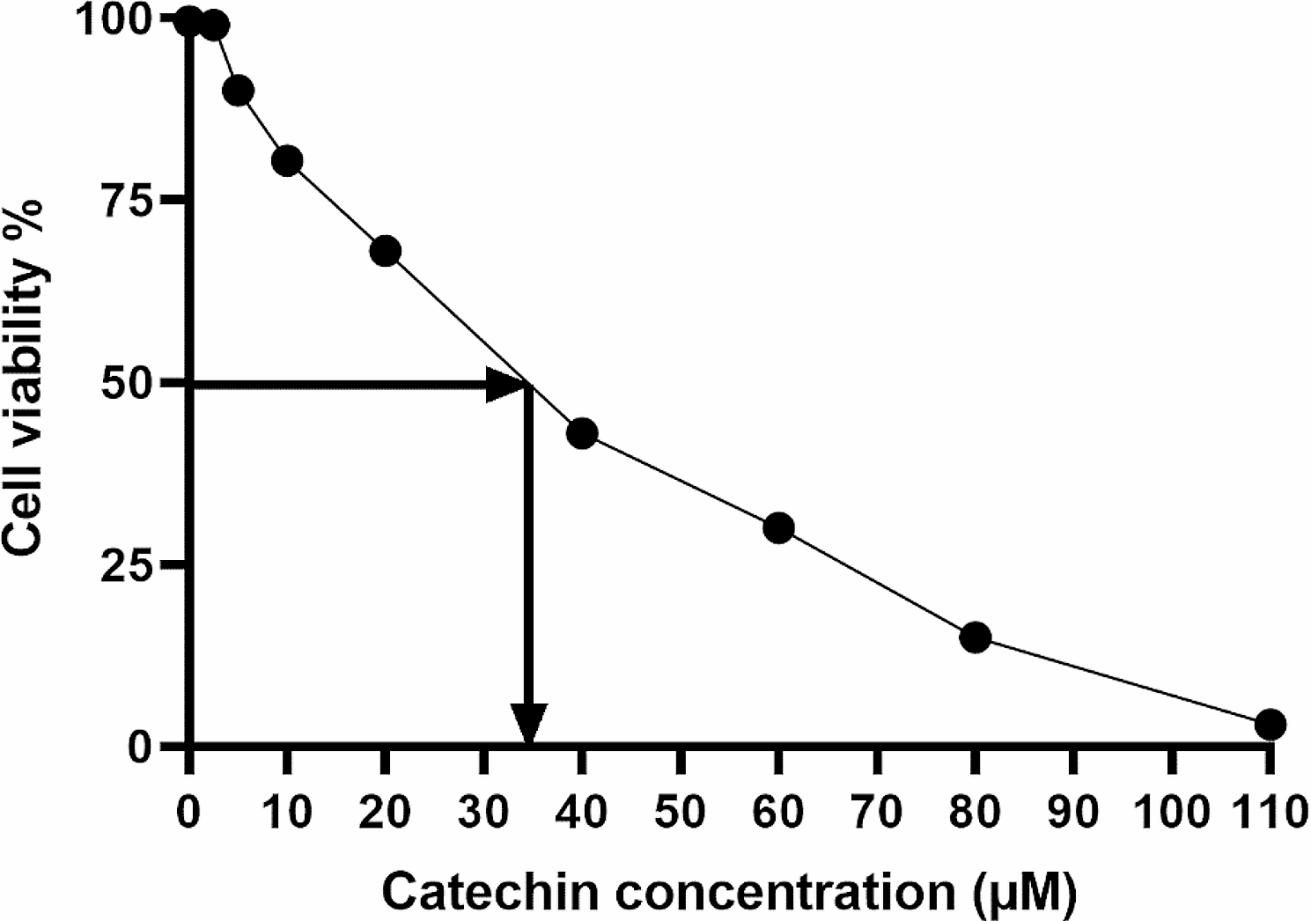
Catechin-induced increase in Nalm6 cell growth and IC50 concentration: Treatment of Nalm6 cells with catechin demonstrated a dose-dependent inhibition of proliferation, with concentrations varying from 0 to 110 µM. The half-maximal inhibitory concentration (IC50) of catechin was established at 35 µM after 24 h, with a 95% confidence interval between 19.5 µM and 39.94 µM. The experimental results showed a robust correlation, evidenced by an R-squared value of 0.941

The use of catechin in Nalm6 cells resulted in significant suppression of cell growth across a range of concentrations, from 0 to 110 After 24 h, the IC50 value of catechin was determined to be 35 µM, with a 95% confidence interval ranging from 19.5 to 39.94. The data obtained from this experiment exhibited a strong correlation with an R-squared value of 0.941, as depicted in Fig. 6.

### Effects of catechin on the morphology and cytoplasm of Nalm6

**Fig. 7:**
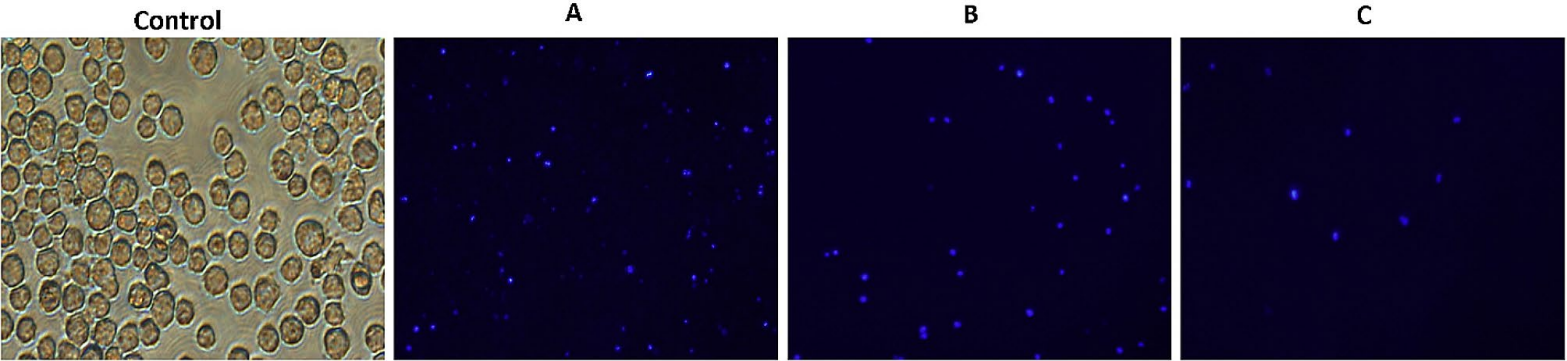
The cytotoxicity and antiproliferative effects of different catechin concentrations (**A**: 10, **B**: 15, and **C**: 20 µM) on Nalm6 cells were detected via DAPI staining at ×100 magnification. Exposure to catechin at concentrations of 10, 15, and 20 µM led to a marked reduction in Nalm6 cell counts within 24 h, particularly at 20 µM. Morphological alterations, including cell aggregation and changes in nuclear size and shape, were observed. DAPI staining indicated increased intercellular distance and signs of chromatin pyknosis as catechin concentration rose. These findings suggest that catechin effectively hampers Nalm6 cell growth and proliferation

With different concentrations of catechin (10, 15, and 20 µM), the cell count started to decrease within 24 h. This decrease was more noticeable at a concentration of 20 µg/ml. Furthermore, the characteristic morphology of the cells gradually changed, resulting in a decrease in cell count and the formation of cell aggregates (Fig. 7). DAPI staining revealed that as the concentration of catechin increased, the distance between the cells also increased, the nuclei became almost larger, chromatin pyknosis became somewhat apparent, the cell shape became rounder, and the nuclei started to fragment. Ultimately, catechin has been demonstrated to inhibit the growth and proliferation of Nalm6 cells.

### Catechin was found to induce apoptosis, as indicated by the flow cytometry data for annexin PI&V

**Fig. 8:**
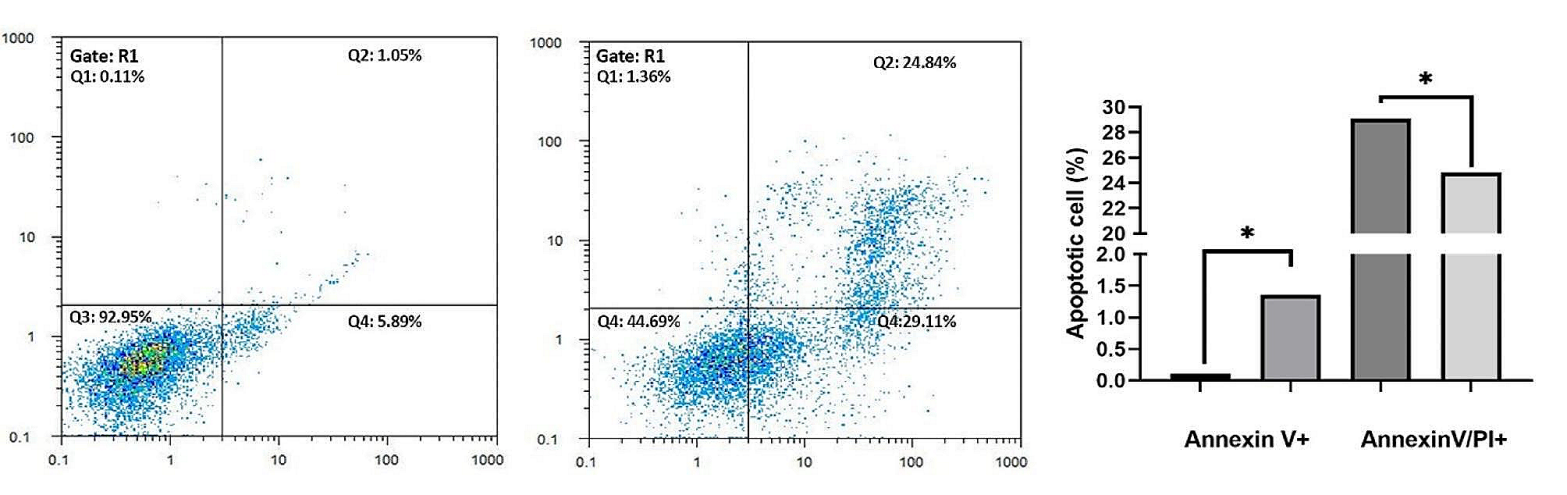
Effect of catechin on the induction of cell death in the Nalm6 cell line. The administered catechin groups showed a significant increase in cell apoptosis (* and **, *P* < 0.05). Compared to those in the untreated group, the concentrations in the catechin group exhibited a significant change of 35 µM in the quarter following the catechin intervention. Specifically, there were changes of 29.11 and 24.84 in the early and late apoptosis quadrants, respectively

As depicted in Fig. 8, catechin has demonstrated a remarkable ability to enhance apoptosis in Nalm6 cells. This enhancement was evident not only in the increased number of annexin V-positive cells but also in the percentage of annexin PI٫V-positive cells. The percentage of annexin V-positive cells increased from 0.11 in untreated cells to 1.05 in treated cells, with a cell treatment IC50 value of 35 µM. Furthermore, compared with those in the untreated group, the catechin concentration in the group treated with catechin exhibited a significant change of 35 µM, representing a quarter of the total catechin concentration in the intervention group. These changes were 29.11% and 24.84% in the early and late apoptosis quadrants, respectively. These findings strongly suggest that catechin exerts its cytotoxic effects on Nalm6 cells by inducing early apoptosis, particularly late apoptosis.

### Prediction of target miRNAs and design of primers, probes, and stem‒Loop sequences

The 3’UTR targets of DNMT3B, DNMT3A, and DNMT1 mRNA can be detected using various websites. More than hundreds of miRNAs confirmed by several miRNA prediction algorithms were selected based on the highest scores. The authors of these studies met several criteria: the number of algorithms, longest seed region, conserved seed region, and simultaneous 3’UTR targeting of DNMT genes; these analyses were not previously performed in the ALL cohort (Tables S1 and S2). Six complementary nucleotides were added to the 3’ ends of the stem‒loop RTs, which were specific for each miRNA. Forward primers and a universal reverse primer were designed along with the TaqMan probe for qPCR. The NCBI Primer-BLAST results for each miRNA revealed that the primer sequences did not bind to any other sequences besides the target miRNA. The results showed 100% specificity for each miRNA (Tables 1 and 2).

### *DNMTs, PODXL*, miR-548, miR-200c, miR-193a, and miR-148a-5p gene expression

**Fig. 9:**
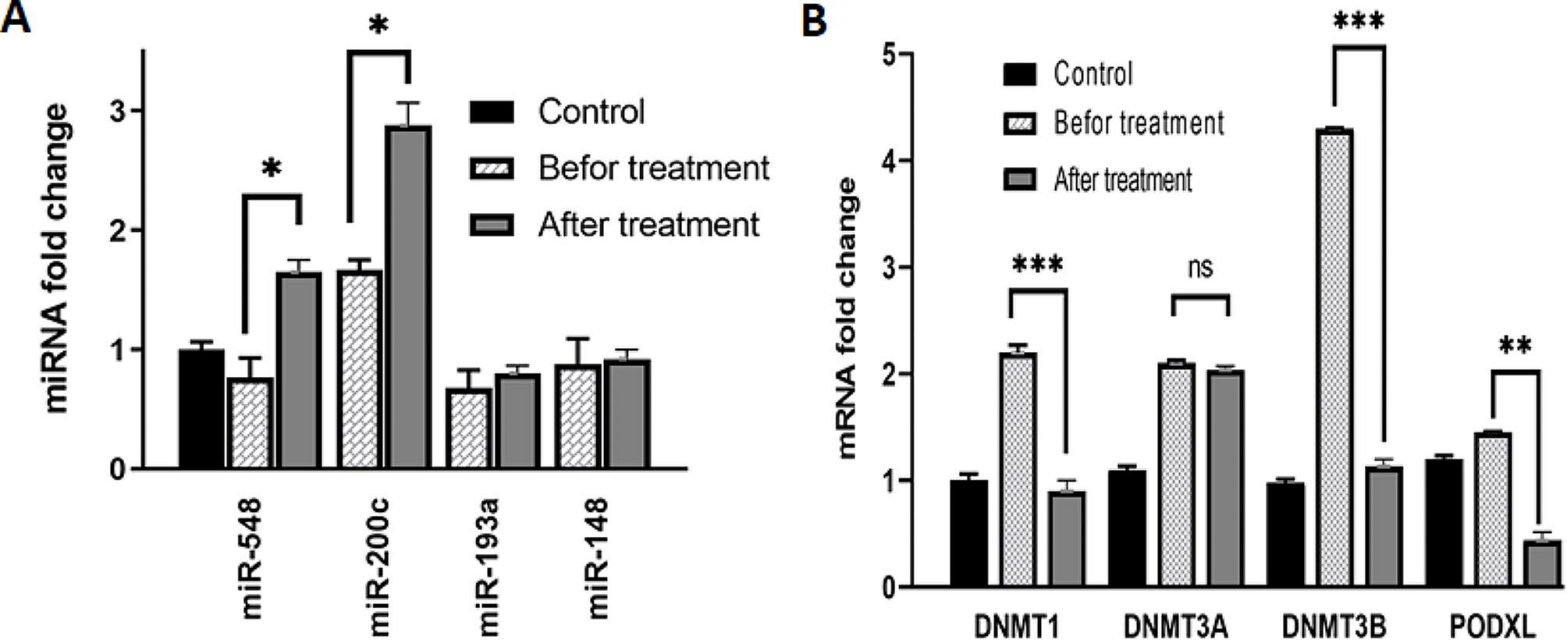
DNMT1, DNMT3A, DNMT3B, miRNA, and PODXL expression in the Nalm6 cell line. The expression of miR-548 and miR-200c increased after treatment with catechin in Nalm6 cells, but the increase in miR-193a and miR-148a-5p was not statistically significant (**A**; p-value > 0.05). Treatment of Nalm6 cells with catechin led to decreased expression levels of DNMT1, DNMT3B, and PODXL compared to those in the untreated cell. However, the decrease in DNMT3A expression was not statistically significant (**B**; p value > 0.05)

In the Nalm-6 cell line, a subset of microRNAs—miR-548, miR-200c, miR-193a, and miR-148a-5p—were identified through computational predictions. Figure 9 illustrates that these miRNAs were expressed at significantly lower levels in Nalm-6 cells compared to untreated cells. Post-catechin treatment, miR-548 and miR-200c levels increased significantly, with fold changes of 1.65 and 2.87, respectively (*p* < 0.05).

PODXL protein, which contributes to cancer progression by interacting with EZR and facilitating cell migration and invasion, was notably overexpressed in Nalm6 cells. This overexpression was substantially reduced following catechin exposure (*p* < 0.05). Concurrently, DNMT1 and DNMT3B expression decreased significantly in catechin-treated Nalm-6 cells relative to untreated cells, as depicted in Fig. 9B. However, the decrease in DNMT3A expression was not statistically significant (*p* > 0.05).

## Discussion

Recent studies have delved into the influence of green tea polyphenols on DNMT enzymes, uncovering their potential to modulate gene expression and DNA methylation—key processes implicated in the oncogenesis and progression of cancer. Our investigation has demonstrated that catechin can effectively bind to the catalytic domains of DNMT enzymes, exhibiting reasonable binding energies. The outcomes of our research indicate that catechin engages in interactions with the catalytic domain of de novo DNMT enzymes, as well as with residues situated in the RFTS and MTase domains of DNMT1. These observations are congruent with prior studies that emphasize the pivotal function of the RFTS domain in replication-dependent DNA methylation and its potential as an auto-inhibitory domain [33–35]. Ndacyayisenga et al. (2024) conducted a comprehensive in silico and in vitro analysis to assess the impact of various catechins, including epigallocatechin (EGC), epicatechin (EC), epigallocatechin gallate (EGCG), and epicatechin gallate (ECG), on gene expression within triple-negative breast cancer (TNBC) cells. Their findings suggest that catechin extracts may facilitate the downregulation of *DNMT1* gene expression in the 4T1 TNBC cell line, proposing an epigenetic mechanism through which green tea polyphenols might exert their anticancer effects [36]. Agarwal’s research in 2023 provided empirical evidence from both in vitro and in vivo models that EGCG could impede tumor proliferation by targeting DNA hypermethylation. The study elucidated that EGCG attenuates DNMT activity and protein levels, encompassing DNMT1, DNMT3a, and DNMT3b, reinstates tumor suppressor genes, and diminishes cell proliferation, thereby offering a multifaceted approach to cancer treatment [37].

In a seminal study by Khan in 2015, the focus was directed towards the effects of (-)-Epigallocatechin-3-gallate (EGCG) on human cervical cancer cells. The results indicated that EGCG could mitigate the activity of both DNMT and histone deacetylases (HDACs), curtail DNMT3B expression, and reactivate tumor suppressor genes (TSGs) due to alterations in promoter methylation [38]. An in vitro examination by Zhi Gao in 2009 revealed that EGCG could demethylate the WIF-1 promoter, reinstating expression and potentially attenuating the Wnt signaling pathway in lung cancer cells [39]. Furthermore, Lee’s investigation in 2005 probed into the mechanisms by which tea catechins and bioflavonoids inhibit DNA methyltransferases. The findings indicated that catechin, epicatechin, and EGCG could inhibit SssI DNMT- and DNMT1-mediated DNA methylation in a dose-dependent manner, with EGCG emerging as the most potent inhibitor [40].

Collectively, these studies underscore the potential of green tea polyphenols, particularly catechins, and EGCG, as epigenetic modulators capable of influencing DNA methylation and gene expression. Conversely, the enzyme methyltransferase can modulate the functionality of miRNA promoter regions through methylation [41]. Our study has revealed that the expression levels of miR-548 and miR-200c were markedly diminished in the leukemia cell line. Nonetheless, post-treatment with catechins, a significant augmentation in the expression of these microRNAs was observed. In contrast, the levels of miR-193a and miR-148a-5p remained relatively unchanged. Various factors could contribute to this observed decline. One plausible mechanism involves alterations in gene expression, particularly through the emergence of single nucleotide polymorphisms (SNPs) within gene regions such as promoters and miRNA sequences [42]. Additionally, the methylation of specific promoter sequences could also constitute a potential underlying factor [42]. In our current study, an upsurge in the expression of the DNMT3Ba and DNMT1 enzymes was detected, leading to aberrant methylation of the miR-548 and miR-200c gene promoters, culminating in their reduced expression.

In the continuation, we observed that the co-administration of catechin leads to a reversal of effects, notably increasing the production of microRNAs (miR-548 and miR-200c) while concurrently decreasing the levels of the DNMT1 and DNMT3B enzyme. This phenomenon underscores the intricate interplay between epigenetic modifications and microRNA expression. Li et al. (2016) explored the epigenetic dysregulation of miR-200c and its participation in a negative feedback loop with DNMT3A in gastric cancer cells. Their study unveiled a novel epigenetic feedback mechanism, where hypermethylation at the miR-200c promoter site resulted in the suppression of miR-200c and the elevation of DNMT3a levels, conversely [43]. Liu et al. (2019) further investigated the role of miR-200b and miR-200c in enhancing the sensitivity of ovarian cancer cells to cisplatin by targeting DNA methyltransferases. Their comprehensive in vitro and in vivo studies indicated that miR-200b and miR-200c potentiate cisplatin sensitivity through the targeting of DNMT3A/B and, indirectly, DNMT1 via the transcription factor Sp1 [44]. To our knowledge, the present study is the inaugural exploration of the effects of catechin treatment on the *PODXL* gene in ALL. The literature delineates a functional association between the *PODXL* gene and miRNAs in various cancers. For instance, miR-199a-5p has been documented to suppress PODXL expression in testicular cancer, suggesting a regulatory role of this microRNA on the gene [42, 45]. Additionally, the inhibition of PODXL in the NT2 cell line has been correlated with reduced invasiveness [42]. The potential indirect interactions between the dysregulation of PODXL, miR-548, and miR-200c, and the methyltransferase genes (DNMT3B and DNMT1) may be attributed to the modulatory effects of these enzymes.

Cheung et al. conducted a parallel assessment of the *PODXL* gene’s role and its interplay with miR-199a in testicular cancer. Their findings indicated that the downregulation of miR-199a led to an upsurge in *PODXL* gene expression, which subsequently enhanced the invasive and migratory capabilities of the cancer cells. Conversely, the suppression of the *PODXL* gene was associated with diminished invasion and migration [42]. Furthermore, the direct influence of miR-5100 on the *PODXL* gene via binding to the 3’UTR has been shown to reduce migration, invasion, and colony formation in pancreatic cancer [46]. The regulation of the *PODXL* gene by the DNMT3B enzyme is plausible, as evidenced by the significant increase in PODXL expression following catechin treatment.

This study pioneers the investigation into the concurrent effects of microRNAs, epigenetic agents (methylation), tumor suppressor genes (*PODXL*), and catechins, which are recognized for their anticarcinogenic properties. In summary, catechins have emerged as promising agents in the realm of cancer therapeutics, highlighting their potential in the modulation of epigenetic and gene expression pathways.

## Conclusion

In conclusion, the findings from our research indicate that catechins possess the ability to inhibit DNMT enzymes. This inhibition correlates with an upregulation of miR-200c and miR-548, alongside a downregulation of the *PODXL* gene expression. The culmination of these molecular events is an enhanced apoptotic response in ALL cells. Our study, therefore, suggests that catechins may offer a promising therapeutic avenue for inducing apoptosis in ALL cells through the modulation of epigenetic and gene expression pathways.

### Electronic supplementary material

Below is the link to the electronic supplementary material.

Supplementary Material 1

Supplementary Material 2

## Data Availability

The datasets generated and/or analyzed during the current study are available in the [NCBI protein database] repository, [https://www.ncbi.nlm.nih.gov/protein/]; [SOPMA server] repository, [https://npsa-prabi.ibcp.fr/NPSA/npsa_sopma.html]; [ProtParam web server] repository, [https://web.expasy.org/protparam/]; [HotSpot Wizard 3] repository, [https://loschmidt.chemi.muni.cz]; [CASTp web server] repository, [http://sts.bioe.uic.edu/castp/index.html?2cpk]; [ADMETlab 2.0 server] repository, [https://admetmesh.scbdd.com/service/evaluation/index]; [YASARA server] repository, [http://www.yasara.org/minimizationserver.htm]. To identify target genes, miRDB, RNAhybrid, PICTAR4, DIANAmT, miRWalk, miRanda, DIANAmT, RNAhybrid, PITA, RNA22, PICTAR5, and TargetScan software were used.
