# Supplementary material for: Catechin-Induced changes in PODXL, DNMTs, and miRNA expression in Nalm6 cells: an integrated in silico and in vitro approach
Source: BMC Complement Med Ther. 2024 Jun 15;24:234. doi: 10.1186/s12906-024-04521-2 (PMC11179370; doi:10.1186/s12906-024-04521-2)
Supplement: Supplementary file 1 — Supplementary Material 1 [file 12906_2024_4521_MOESM1_ESM.docx]

**Table S1**: Prediction of target miRNAs based on the number of software’s

| **Gene** | **MicroRNA** | **miRanda** | **miRDB** | **miRWalk** | **RNAhybrid** | **PICTAR4** | **PICTAR5 PITA** | **RNA22** | **Targetscan** | **SUM** |
| --- | --- | --- | --- | --- | --- | --- | --- | --- | --- | --- |
| [DNMT3B](http://www.ncbi.nlm.nih.gov/sites/entrez?Db=gene&Cmd=ShowDetailView&TermToSearch=1789&ordinalpos=1&itool=EntrezSystem2.PEntrez.Gene.Gene_ResultsPanel.Gene_RVDocSum) | [hsa-miR-548](http://microrna.sanger.ac.uk/cgi-bin/sequences/mirna_entry.pl?acc=MI0000105) | 1 | 1 | 1 | 0 | 1 | 0 | 0 | 1 | 5 |
| DNMT3B | hsa-miR-200c | 0 | 1 | 1 | 0 | 0 | 0 | 0 | 1 | 3 |
| DNMT3B | hsa-miR-193a | 0 | 1 | 1 | 0 | 1 | 0 | 0 | 1 | 4 |
| DNMT3B | hsa-miR-148 | 0 | 0 | 1 | 0 | 1 | 0 | 0 | 1 | 3 |
| DNMT3A | [hsa-miR-548](http://microrna.sanger.ac.uk/cgi-bin/sequences/mirna_entry.pl?acc=MI0000105) | 0 | 1 | 1 | 0 | 1 | 0 | 0 | 1 | 4 |
| DNMT3A | hsa-miR-200c | 0 | 0 | 1 | 0 | 1 | 0 | 0 | 1 | 3 |
| DNMT3A | hsa-miR-193a | 0 | 1 | 1 | 0 | 1 | 0 | 0 | 1 | 4 |
| DNMT3A | hsa-miR-148 | 1 | 0 | 1 | 0 | 0 | 0 | 0 | 1 | 3 |
| DNMT1 | hsa-miR-548 | 0 | 1 | 0 | 1 | 0 | 0 | 0 | 1 | 3 |
| DNMT1 | hsa-miR-200c | 0 | 1 | 1 | 0 | 0 | 0 | 0 | 1 | 3 |
| DNMT1 | hsa-miR-193a |  | 1 | 1 | 0 | 0 | 1 | 0 | 1 | 4 |
| DNMT1 | hsa-miR-148 |  | 1 | 1 | 0 | 0 | 0 |  | 1 | 3 |

**Table S4:** Prediction of target miRNAs based on gene region and seed length

| **Gene Name** | **Ref Seq ID** | **MicroRNA** | **Seed Length** | **3^'^UTR Length** | **Region** |
| --- | --- | --- | --- | --- | --- |
| *DNMT3B* | NM_006892 | hsa-miR-548P | 12 | 4256 | 3'UTR |
| *DNMT3B* | NM_006892 | hsa-miR-200c | 10 | 4256 |  |
| *DNMT3B* | NM_006892 | hsa-miR-193a | 8 | 4256 |  |
| *DNMT3B* | NM_006892 | hsa-miR-148 | 8 | 4256 |  |
| *DNMT3A* | NM_175629 | hsa-miR-548P | 9 | 3876 | 3'UTR |
| *DNMT3A* | NM_175629 | hsa-miR-200c | 7 | 3876 |  |
| *DNMT3A* | NM_175629 | hsa-miR-193a | 7 | 3876 |  |
| *DNMT3A* | NM_175629 | hsa-miR-148 | 10 | 3876 |  |
| *DNMT1* | NM_0013082 | hsa-miR-548P | 8 | 5269 | 3'UTR |
| *DNMT1* | NM_0013082 | hsa-miR-200c | 7 | 5269 |  |
| *DNMT1* | NM_0013082 | hsa-miR-193a | 7 | 5269 |  |
| *DNMT1* | NM_0013082 | hsa-miR-148 | 8 | 5269 |  |
