# Supplementary material for: Catechin-Induced changes in PODXL, DNMTs, and miRNA expression in Nalm6 cells: an integrated in silico and in vitro approach
Source: BMC Complement Med Ther. 2024 Jun 15;24:234. doi: 10.1186/s12906-024-04521-2 (PMC11179370; doi:10.1186/s12906-024-04521-2)
Supplement: Supplementary file 2 — Supplementary Material 2 [file 12906_2024_4521_MOESM2_ESM.docx]

This supplementary file contains the R code used to perform the Absorption, Distribution, Metabolism, Excretion, and Toxicity (ADMET) analysis of the studied compounds using the ADMETLAB software package. The code demonstrates the step-by-step process of loading the required libraries, importing the compound data, and running the ADMET predictions. It also includes the necessary data preprocessing steps, such as handling missing values and formatting the input data to meet the software's requirements. The code is well-documented with comments explaining each section's purpose and functionality. This file serves as a reference for reproducing the ADMET analysis and ensuring the transparency of the computational methods employed in the study.

#Load the file

Admet_list=read.csv('C:/Users/ExpertSystems/Desktop/newproject/man/mining/mining.csv',header=T,sep=',')

#Load the necessary library

library(dplyr)

#PhysicochemicalProperty

drug<-mutate(Admet_list,mw=ifelse(MW>=100&MW<=600,1,0))

drug<-mutate(drug,nha=ifelse(nHA>=0&nHA<=12,1,0))

drug<-mutate(drug,nhd=ifelse(nHD>=0&nHD<=7,1,0))

drug<-mutate(drug,nrot=ifelse(nRot>=0&nRot<=11,1,0))

drug<-mutate(drug,nring=ifelse(nRing>=0&nRing<=6,1,0))

drug<-mutate(drug,maxring=ifelse(MaxRing>=0&MaxRing<=18,1,0))

drug<-mutate(drug,nhet=ifelse(nHet>=1&nHet<=15,1,0))

drug<-mutate(drug,fchar=ifelse(fChar>=-4&fChar<=4,1,0))

drug<-mutate(drug,nrig=ifelse(nRig>=0&nRig<=30,1,0))

drug<-mutate(drug,flexibility=ifelse(Flex>=0&Flex<1.83,1,0))

drug<-mutate(drug,nstereo=ifelse(nStereo<=2,1,0))

drug<-mutate(drug,tpsa=ifelse(TPSA>=0&TPSA<=140,1,0))

drug<-mutate(drug,logs=ifelse(LogS>=-4&LogS<=0.5,1,0))

drug<-mutate(drug,logp=ifelse(LogP>=0&LogP<=3,1,0))

drug<-mutate(drug,logd=ifelse(LogD>=1&LogD<=3,1,0))

#MedicalChemistry

drug<-mutate(drug,qed=ifelse(QED>0.67,1,0))

drug<-mutate(drug,synth=ifelse(Synth<=6,1,0))

drug<-mutate(drug,fsp3=ifelse(Fsp3>=0.42,1,0))

drug<-mutate(drug,mce.18=ifelse(MCE.18>=45,1,0))

drug<-mutate(drug,natural.product.likeness2=ifelse(Natural.Product.likeness>=0&Natural.Product.likeness<=5,1,0))

drug<-mutate(drug,LipinskiRule=ifelse(Lipinski=='Accepted',1,0))

drug<-mutate(drug,PfizerRule=ifelse(Pfizer=='Accepted',1,0))

drug<-mutate(drug,GSKRule=ifelse(GSK=='Accepted',1,0))

drug<-mutate(drug,goldentriangle=ifelse(GoldenTriangle==0,1,0))

drug<-mutate(drug,pains=ifelse(PAINS==0,1,0))

drug<-mutate(drug,ALARMNMRRule=ifelse(Alarm_NMR==0,1,0))

drug<-mutate(drug,BMSRule=ifelse(BMS==0,1,0))

drug<-mutate(drug,ChelatorRule=ifelse(Chelating==0,1,0))

#Absorption

drug<-mutate(drug,caco.2=ifelse(Caco.2>-5.15,1,0))

drug<-mutate(drug,mdck=ifelse(MDCK>0.000002,1,0))

drug<-mutate(drug,pgp.inh=ifelse(Pgp.inh>=0&Pgp.inh<0.3,1,0))

drug<-mutate(drug,pgp.sub=ifelse(Pgp.sub>=0&Pgp.sub<0.3,1,0))

drug<-mutate(drug,hia=ifelse(HIA>=0&HIA<0.3,1,0))

drug<-mutate(drug,F20=ifelse(F.20..>=0&F.20..<0.3,1,0))

drug<-mutate(drug,F30=ifelse(F.30..>=0&F.30..<0.3,1,0))

#Distribution

drug<-mutate(drug,ppb=ifelse(PPB<=0.90,1,0))

drug<-mutate(drug,vdss=ifelse(VDss>=0.04&VDss<20,1,0))

drug<-mutate(drug,bbb=ifelse(BBB>=0&BBB<0.3,1,0))

drug<-mutate(drug,fu=ifelse(Fu>=0.05,1,0))

#Metabolism

drug<-mutate(drug,cyp1a2.inh=ifelse(CYP1A2.inh==0,1,0))

drug<-mutate(drug,cyp2c19.inh=ifelse(CYP2C19.inh==0,1,0))

drug<-mutate(drug,cyp2c9.inh=ifelse(CYP2C9.inh==0,1,0))

drug<-mutate(drug,cyp2d6.inh=ifelse(CYP2D6.inh==0,1,0))

drug<-mutate(drug,cyp3a4.inh=ifelse(CYP3A4.inh==0,1,0))

drug<-mutate(drug,cyp1a2.sub=ifelse(CYP1A2.sub==1,1,0))

drug<-mutate(drug,cyp2c19.sub=ifelse(CYP2C19.sub==1,1,0))

drug<-mutate(drug,cyp2c9.sub=ifelse(CYP2C9.sub==1,1,0))

drug<-mutate(drug,cyp2d6.sub=ifelse(CYP2D6.sub==1,1,0))

drug<-mutate(drug,cyp3a4.sub=ifelse(CYP3A4.sub==1,1,0))

#Excretion

drug<-mutate(drug,cl=ifelse(CL>=5,1,0))

drug<-mutate(drug,t1.2=ifelse(T12>=0&T12<0.3,1,0))

#Toxicology

drug<-mutate(drug,herg=ifelse(hERG>=0&hERG<0.3,1,0))

drug<-mutate(drug,h.ht=ifelse(H.HT>=0&H.HT<0.3,1,0))

drug<-mutate(drug,dili=ifelse(DILI>=0&DILI<0.3,1,0))

drug<-mutate(drug,ames=ifelse(Ames>=0&Ames<0.3,1,0))

drug<-mutate(drug,roa=ifelse(ROA>=0&ROA<0.3,1,0))

drug<-mutate(drug,fdamdd=ifelse(FDAMDD>=0&FDAMDD<0.3,1,0))

drug<-mutate(drug,skinsen=ifelse(SkinSen>=0&SkinSen<0.3,1,0))

drug<-mutate(drug,carcinogenicity=ifelse(Carcinogenicity>=0&Carcinogenicity<0.3,1,0))

drug<-mutate(drug,ec=ifelse(EC>=0&EC<0.3,1,0))

drug<-mutate(drug,ei=ifelse(EI>=0&EI<0.3,1,0))

drug<-mutate(drug,respiratory=ifelse(Respiratory>=0&Respiratory<0.3,1,0))

drug<-mutate(drug,bcf=ifelse(BCF>=0&BCF<1,1,0))

drug<-mutate(drug,igc50=ifelse(IGC50>7&IGC50<=10,1,0))

drug<-mutate(drug,lc50fm=ifelse(LC50>7&LC50<=10,1,0))

drug<-mutate(drug,lc50dm=ifelse(LC50DM>7&LC50DM<=10,1,0))

drug<-mutate(drug,NR.AR2=ifelse(NR.AR>=0&NR.AR<0.3,1,0))

drug<-mutate(drug,NR.AR.LBD2=ifelse(NR.AR.LBD>=0&NR.AR.LBD<0.3,1,0))

drug<-mutate(drug,NR.AhR2=ifelse(NR.AhR>=0&NR.AhR<0.3,1,0))

drug<-mutate(drug,NR.Aromatase2=ifelse(NR.Aromatase>=0&NR.Aromatase<0.3,1,0))

drug<-mutate(drug,NR.ER2=ifelse(NR.ER>=0&NR.ER<0.3,1,0))

drug<-mutate(drug,NR.ER.LBD2=ifelse(NR.ER.LBD>=0&NR.ER.LBD<0.3,1,0))

drug<-mutate(drug,NR.PPAR.gamma2=ifelse(NR.PPAR.gamma>=0&NR.PPAR.gamma<0.3,1,0))

drug<-mutate(drug,SR.ARE2=ifelse(SR.ARE>=0&SR.ARE<0.3,1,0))

drug<-mutate(drug,SR.ATAD52=ifelse(SR.ATAD5>=0&SR.ATAD5<0.3,1,0))

drug<-mutate(drug,SR.HSE2=ifelse(SR.HSE>=0&SR.HSE<0.3,1,0))

drug<-mutate(drug,SR.MMP2=ifelse(SR.MMP>=0&SR.MMP<0.3,1,0))

drug<-mutate(drug,SR.p53.2=ifelse(SR.p53>=0&SR.p53<0.3,1,0))

drug<-mutate(drug,Acute_Aquatic_ToxicityRule=ifelse(Acute_Aquatic_Toxicity==0,1,0))

drug<-mutate(drug,Genotoxic_CarcinogenicityRule=ifelse(Genotoxic_Carcinogenicity_Mutagenicity==0,1,0))

drug<-mutate(drug,NonGenotoxic_CarcinogenicityRule=ifelse(NonGenotoxic_Carcinogenicity==0,1,0))

drug<-mutate(drug,ld50_oral=ifelse(LD50_oral>=0,1,0))

drug<-mutate(drug,Skin_SensitizationRule=ifelse(Skin_Sensitization==0,1,0))

drug<-mutate(drug,NonBiodegradableRule=ifelse(NonBiodegradable==0,1,0))

drug<-mutate(drug,SureChEMBLRule=ifelse(SureChEMBL==0,1,0))

drug<-mutate(drug,Toxicophores2=ifelse(Toxicophores==0,1,0))

#......................................................

main=select(drug,mw:Toxicophores2)

Catechin=sum(main[1,])
